# The Living-Out System at Leavesden

**Published:** 1913-03-29

**Authors:** 


### THE LIVING-OUT SYSTEM AT LEAVESDEN.

### Dr. F. A. Elkins on the Theory and Practice of the Scheme.

The mental hospital world of late has been pushing to the front more than one of those problems which have been so long discussed, and the solution of which has been so much delayed—for instance, the status of the assistant medical officer in mental hospitals. The institutional atmosphere which prevents him from marrying, binds him at all hours to the hospital, limits his intercourse even with his colleagues in the same branch of administrative medicine—in short, which sacrifices his life on the altar of institutional discipline; all this is now being actively discussed with a view to a more satisfactory rearrangement of personal liberty and institutional claims. But the assistant medical officer is not the only person in the mental hospital on whom the institutional system, as at present enforced, weighs heavily. The attendant, male and female, is in a parallel case, with this all-important difference, however, that in their case an experiment on freer lines has been actually in force on a developing scale at, at least, one mental hospital. With a view, therefore, of throwing some light on parallel problems, especially to that presented by proper relation of discipline and personal freedom in the attendant's case, our representative visited Leavesden Mental Hospital, King's Langley, in order to hear from Dr. F. A. Elkins, the Medical Superintendent, and the principal advocate of the living-out system for mental hospital attendants and nurses, the origin, theory, and practice of this still debated administrative reform.

### How Objections are Met.

"May I ask what first turned your attention to the advocacy of the living-out system?"

"Like most ideas which are destined to fructify it grew slowly as my attention was more and more drawn to considering the best method of dealing with the complaints, and the actual causes which found a vent in them, that beset the senior residents and superintendents in every mental hospital. Some of the latter have a short way of dealing with them. They dismiss the complaining officials. But complaints, or rather grumblings, at the food and institutional restraints of the mental hospital attendant's life are too numerous to be treated thus summarily, and it occurred to me, as it has occurred to others, to see if it were possible to find a principle which would meet the difficulties at their source. When I was working under Sir Thomas Clouston at Edinburgh I remember observing, what afterwards struck me in this connection, that the stewards in some Scottish mental hospitals live out, while it was considered when I entered on administrative duties in English institutions that here they could never be allowed to do so. It answered in Scotland; why could it not answer here? That was one of the early questions which I tried to answer."

"Proposals for adopting the living-out system at first received objections?"

"At first, certainly; but what were these? It was said that unless the staff of attendants and other officials were under institutional restraint there could be no adequate discipline. But institutional restraint was everywhere one of the causes of grumbling. It was said that punctuality could never be enforced if the staff lived out. But leave to live out means permission to marry, and for this long-wanted privilege it seemed, and has proved, that the price of punctuality will be willingly paid. It was said that if money were given in lieu of food, as would be necessary did the staff board out, food would be stolen. But the institution food, though very liberal almost everywhere, was a universal cause of complaint. Might not the cause be removed if the men were allowed to order whatever their appetites fancied in their own homes? For on the dietetic fancies of mental staffs there is this to be said. The nature of their work, the sights and sounds to which they are subjected, the subconscious strain which their responsibilities entail—all these tend to have a deleterious effect upon digestion; and, consequently, with the lengthy hours of work and the amount of time spent in or under the shadow of the institution, produce a jaded appetite. This is the effect of their work, for which sympathy and treatment rather than blame is indicated. As to stealing, the routine precautions are sufficient to guard against that, though in so large a community a thief may be found at times. Two further objections were urged. The first was that those who lived out would bring infectious diseases into the institution. This is easily answered: first, by the fact that the class from which the attendant is drawn is a very respectable one, for his status has greatly improved, and permission to marry offers an attraction to many of the best men who drift out of the service where this is withheld; and, secondly, that the highly healthy surroundings and homes in which they live practically preclude the possibility of their introducing infection; or, at least, make them far less liable to do so than are the visitors to the patients, many of them from the slums of our cities, who are not precluded on this account. But the first objections that I mentioned—the absence of discipline, the fears of unpunctuality, the temptation to steal—are the more interesting, for they provide the key to the whole of our contentions here as to the advantages of letting as many of the staff live out as possible. The food, the institutional discipline, the punctuality (secured by the living-in system), and the inability to marry consequent upon the two latter: these were the very causes of the complaints. If we removed the causes we should do away with the complaints. And that is what has happened. Logically, therefore, it seems to me that those who object to our living-out system on the above grounds, which are those invariably urged, are supporting the causes of complaint which all superintendents admit to be general, similar, and difficult to deal with. The above is the theoretical basis on which the living-out system rests."

"In the case of the nurses, have not moral objections been made?"

"Yes; and not in regard to them only. Some have urged the necessity of mental hospital authorities looking after the lives and morals of the staff when off duty, and these good people have explained that this is why officials should not be allowed to live out. The answer to this is simply that it is impossible. Those who want to go wrong do so in spite of any precautions that may be taken. No outside power can prevent them from doing so. But such persons should not be in the service. We quite admit also that in an individual case now and again we have met with a difficulty of this kind. But this difficulty is proportionately greater among the in-living, and so does not affect the value of the living-out system."

"Who live out at present?"

"A distinction must be drawn between those who are provided with cottages by the Board on the hospital estate and those who are permitted to live beyond the estate in rooms or houses, which, of course, have to be approved. Certain officials must always live on the estate; such are the medical superintendent, though his house should have its own access to the public highway; the chief engineer, who is also captain of the fire-brigade; and the matron. I have said that I do not think that it is necessary for the steward to do so, and I may add that, provided arrangements are made for the senior assistant medical officer to have rooms in the hospital whenever the medical superintendent is away, I do not see why the former should be compelled to live in the institution. At Leavesden he is married, and has a house on the fringe of the estate. However, taking the figures which I published a year or two ago as a low average, out of eighty-two day nurses fifty-eight boarded and lodged out of the institution, all the nineteen night attendants did so, while, on the other hand, only twenty-two out of ninety-six day nurses lodged and boarded out. The reason of this is not that living out is a bad system for women—too many village nurses, Queen's nurses, to say nothing of village dressmakers, schoolmistresses, and countless others do so without reproach for us to have doubts about that; but that as a nurses' home has been built, it would be foolish not to occupy it, unless the day comes when the home can be converted into a villa-residence for patients of a suitable kind, paying a moderate board, who form a class of the community badly provided for in England."

### The Practical Advantages of the System.

"Apart from theoretical, what have been the practical advantages of the living-out system?"

"It has, first, undoubtedly increased the length of service of all classes of the attendants. In the mental hospital's report published last year I was able to state that of one hundred and ten male attendants of all ranks three only had less than one year's service; no head, charge, night, or deputy-charge attendant had less than five years' service, while on the laundry female staff the head laundress and her deputy live in, while the other twelve live out, and six of these have over five years' service. The second advantage has been a gain and save in wages—a gain to the Asylums Board because the money given in lieu of rations does not quite represent the actual cost of feeding each member in the institution; and a gain to the attendants, since these—and they are the majority—are enabled to give the extra money to their wives, which is regarded as a very useful addition to the salaries. Thirdly, it has allowed more beds to be placed at the disposal of patients without the expense of erecting new buildings, as attendants' rooms have been converted to patients' purposes. This will also mean, if the living-out system be regularly extended, great economies in the future building of staff quarters. These are the three chief advantages from the Board's and the superintendent's points of view. From that of the attendants' it has given at one stroke a better atmosphere in place of the 'asylum atmosphere,' which is so pernicious after a series of years to those subjected to it; and it has made family life possible. The husbands now at home for meals see their wives and know their children. Previously only one day a week was spent at home. This family atmosphere outside the institution has also tended to counteract the undoubted dangers which affect those who spend a long life in the company of the insane. These dangers are disputed, and statistics of 1 per cent. of insanity among mental hospital attendants are pointed at in proof. The answer is that the class from which the attendants are drawn is one of good physique, for the work demands it; and, secondly, that such statistics take no count of the numerous breakdowns and departures from the service with which every medical officer is but too well acquainted."

### Should Cottages be Built?

"You would have cottages on the estate for attendants, then?"

"I thought so once; but I prefer now to let the local demand for lodgings for those who live out to create its supply. The twenty-two cottages which the Board built some years ago showed the disadvantages of building. First, they were on the estate, and still close to, if not in, the institution atmosphere; secondly, from economy they were built in rows, so that the neighbours of those who lived in them were engaged in the same pursuits—so the object of the cottages was in some degree negatived; lastly, no Board can build as cheaply as the local builder, and why should it attempt to do so when, by supporting a demand in allowing the staff to live out, the local builder will be only too anxious to supply the cottages? That, at least, has been the experience here."

"Did you foresee the building of these cottages in the locality?"

"Not exactly, for one of the most remarkable things in the development of the living-out system has been the way in which the attendants and nurses—especially the latter—have, on living out, attracted to the neighbourhood their relatives and friends with whom to live. It began in August 1905. The first charge-nurse went out to live with her mother. Since then more than one family related to the members of the staff has migrated here. Hence the demand for cottages."

"Could the Leavesden ideas be applied to all mental hospitals?"

"I am bound to admit that our situation between two villages is an extremely fortunate one, and that all other institutions are not so fortunately situated. Some are in towns; but then the acknowledged principle is that mental hospitals should be placed in rural surroundings, and where they are so placed, as in the case of almost every new one now building or to be built, the difficulty of allowing living out in a town will disappear. So that the criticism involved here is rather one of the site of the town hospital than of the principle of living out itself."

